# MSI expresso: a software for determining MSI status and detecting MSI-related transcription events from RNA sequencing data

**DOI:** 10.3389/fgene.2025.1523278

**Published:** 2025-04-24

**Authors:** Emmanuel Tubacher, Alexandre How-Kit, Mourad Sahbatou, Alex Duval, Victor Renault, Jean-François Deleuze

**Affiliations:** ^1^ Laboratory for bioinformatics, Fondation Jean DAUSSET Centre d’Etude du Polymorphisme Humain, Paris, France; ^2^ Laboratory for Genomics, Fondation Jean Dausset Centre d'Etude du Polymorphisme Humain, Paris, France; ^3^ Centre de recherche Saint-Antoine, Inserm UMR_S 938, équipe “instabilité des microsatellites et cancers”, Paris, France

**Keywords:** microsatellite instability, cancer, rnaseq, exon skipping, circos, UTR

## Abstract

Summary: Microsatellite instability (MSI) is becoming increasingly important in oncology as it has been reported across more than two dozen of solid cancer types. The MSI-high phenotype has long been used as a predictive and prognostic marker in colorectal cancer and has been recently approved by the FDA as a marker for immune checkpoint blockade therapy for solid cancers. Several bioinformatics tools have been developed to assess MSI status of a tumor sample using Next-Generation Sequencing (NGS) data mostly from whole genome, whole exome, and targeted gene sequencing data. While most tools available only infer the MSI status, none of them use RNA-sequencing (RNA-seq) data and provide per microsatellite expression and genotype results. We present MSI Expresso, a software which assesses the MSI status by testing the instability of a panel of 3′UTR microsatellites from RNA-seq data and also provides a detailed landscape of MSI-related events such as exon skipping, unstable coding and intronic microsatellites with a graphical output of the recurrent events. MSI Expresso’s ability to detect the MSI status was assessed from RNA-seq data of 228 colon, 13 prostate and two endometrial cancer samples with known MSI status and achieved almost 100% concordant results. Thus, MSI Expresso is a new tool for MSI detection from RNA-sequencing data complementary to genomic and genetic approaches allowing to explore the consequence of MSI events on transcripts/transcriptome.

## Background

**FIGURE 1:**
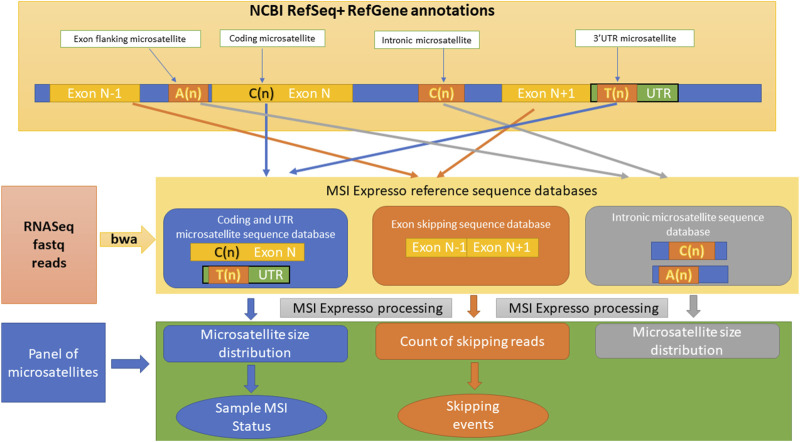
Overview of the different steps included in the MSI Expresso algorithm.

Microsatellites, also known as simple sequence repeats, are commonly defined as DNA sequences of variable numbers (at least 5) of a repeated unit composed of one–six nucleotides (Fan and Chu, 2007). The MSIExpresso pipeline is shown in Figure 1. Microsatellite instability (MSI) is defined as a hypermutable phenotype caused by the loss of DNA mismatch repair (MMR) activity (Boland and Goel, 2010). It has been long associated with many cancers, including notably colorectal (CRC) and endometrial cancers (Kim et al., 2013) and has since been reported in 27 cancer types (Bonneville et al., 2017). Besides, FDA recently approved immune checkpoint therapy for patients with a MSI-high (MSI-H) solid tumor (Marcus et al., 2019).

As a consequence, and with the extraordinary increase of Next-Generation Sequencing (NGS) data available, several tools have been developed to infer the MSI status of a tumor sample from NGS raw data mainly based on whole-genome (WGS), whole exome (WES) and targeted gene sequencing (TGS) experiments. Notably, it includes MANTIS (Bonneville et al., 2017) and MSIsensor (Niu et al., 2013), among others (Baudrin et al., 2018; Renault et al., 2022). However, to our knowledge only three algorithms have been described to date for RNA-seq data: the first is based on the global detection of insertions and deletions in microsatellites found in expressed genes (Danaher et al., 2019; Lu et al., 2013), the second uses the expression level of MMR genes to predict the MSI status (Danaher et al., 2019), while the last one redefines a gene signature combining already published MSI gene signatures derived from microarray expression data and a new gene signature extracted from TCGA colon cancer gene expression data (Pačínková and Popovici, 2019). Additionally, RNA-sequencing data allows the study of exon skipping, a post-transcriptional event that is particularly interesting in the context of MSI cancer. Thus, the instability of microsatellites close to the exon acceptor sites (A or T homopolymers) can lead to the skipping of exons during the splicing process, producing premature termination codons (PTS) or truncated proteins like in *HSP110* gene (Buhard et al., 2016; Dorard et al., 2011). Additionally, GC-rich intronic expansions have been associated with intron retention (Sznajder et al., 2018). Finally, coding microsatellites instability usually produces frameshifts and introduces PTS in the coding sequence like in *ACVR2A*, *BAX* and *TGFβR2* genes (Cortes-Ciriano et al., 2017). Recently, this approach has been applied to RNA-seq data from colonic samples, including mucosa, adenoma and tumors, and allowed the identification of key driver MSI events in the pathogenesis of colorectal MSI cancer (Jonchère et al., 2024). The estimation of these events from RNA-Seq data should reveal the true impact of coding and intronic MSI upon transcription and gene expression and might give a more refined overview of genomic and transcriptomic alterations present in tumor samples in addition to the MSI status.

In this context, we have developed MSI Expresso, a complete ready-to-use bioinformatic workflow allowing raw data analysis to graphical representation, which infers the MSI status from RNA-seq data by testing the instability of a panel of 3′UTR microsatellites. The software also provides a detailed landscape of MSI-related events such as exon skipping, intronic and coding microsatellites instability, and the commonly associated mismatch repair deficiency (MMRd) status estimation, which might highlight events with functional consequences in the tumor. Our software might thereby be useful both for basic and translational applications in oncology.

## Materials and methods

### Publicly available NGS RNAseq cancer data used for MSI expresso assessment

MSI Expresso was developed using the RNASeq MICROSPLICOTHER dataset EGAS00001004863, 101bp paired-end sequencing RNAseq, composed of:

−33 CRC normal/tumoral MSS pairs
−90 CRC normal/tumoral MSI pairs
−5 MSI cell lines (Co115, HCT8, HCT116, Lim1215 and LS174T)
−5 MSS cell lines (Caco2, HT29, LS173, SW480 and SW620)

MSI Expresso was tested on the following freely-available datasets:

−77 gastric cancer samples (73 MMS and 4 MSI) from the ERP010795 Korean Study 101-bp paired-end sequencing RNASeq
−37 gastric cancer samples (26 MSS and 11 MSI) from the SRP014574 Korean Study, 90-bp paired-end sequencing RNASeq
−10 LnCAP (prostate) MSI cancer cell line sample from SRP120165 study, 76-bp and 151 bp single-end RNAseq
−4 VCaP MSS prostate cancer cell line samples from SRP132915 study, 76-bp single-end RNASeq
−3 LnCAP MSI cancer cell line samples from SRP132915 study, 76-bp single-end RNASeq
−2 MSS prostate cancer samples, 3 MSI prostate samples, two endometrium MSI samples and two colon MSI samples from SRP186687 study, 101-bp paired-end RNASeq
−12 22RV1 MSI prostate cancer samples from SRP187530 study, 51-bp paired-end RNASeq
−15 colon cancer samples (7 MSS and 8 MSI with one replicate) from the PRJNA784142 study, 150-bp paired-end RNASeq
−6 MSI endometrial cancer samples from SRP320041 study, 150-bp paired-end RNASeq
−10 MSS endometrial cancer samples from SRP251211 study, 50-bp single-end RNASeq
−1 MSI endometrial cancer sample from SRP217942 study, 150-bp paired-end RNASeq
−2 MSI endometrial cancer samples from SRP186687 study, 101-bp paired-end RNASeq
−34 paired normal/tumoral hepatocellular samples from SRP338575 study 150-bp paired-end RNASeq as negative control for MSI and MMR status and false positive rate estimation.

### Algorithm implementation

#### Source codes and computer requirements

**FIGURE 2:**
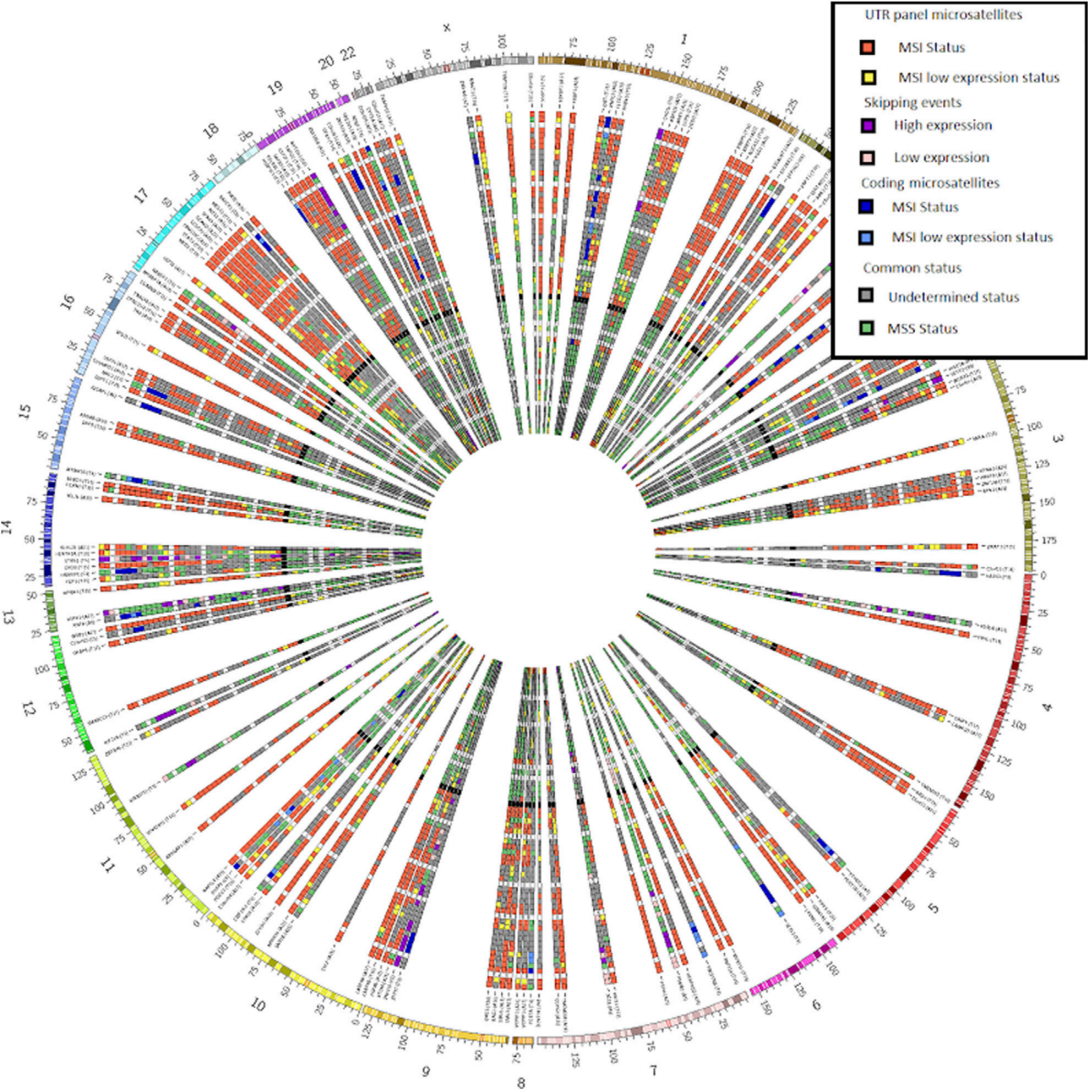
CIRCOS plot from our bundled tool MSI Expresso2Circos showing the inferred MSI status on the UTR microsatellite panel used to determine the MSI status (red and yellow tiles), some coding microsatellites (dark and clear blue tiles) and exon skipping events (purple and pink tiles) for a selection of MSI or MSS samples from various cancers.

MSI Expresso is written in perl, runs under linux and only requires widely used bioinformatic tools: bwa, bcftools and samtools (Li and Durbin, 2009) for the analysis part and CIRCOS (Krzywinski et al., 2009) for the graphical output (Figure 2). MSI Expresso is freely available at https://github.com/FJD-CEPH/MSIExpresso.

We run MSI Expresso on a Linux machine with 16 CPUs and 48 Go of RAM, the main memory/CPU usage being made by the BWA aligner running with 16 threads. In this configuration, each complete analysis takes less than an hour for a 60 M reads paired ends sample (coding, intronic and exon-junction microsatellites, exon skipping event, MMR status estimation). Reducing the number of the threads will of course increase the computation time, but any Linux machine with at least 8 GB of RAM should be able to complete an MSI Expresso run in few hours. In order to reduce computation time in clinical context, we provide a streamlined version of MSIExpresso analysis that assesses MSI status from a selected panel of microsatellites and optionally estimates MMR status.

In this study, we aimed to develop a bioinformatic tool, MSI Expresso, allowing both the determination of the MSI status, the MMRd status and the detection of MSI-related transcription events using RNA-seq data from tumor samples. MSI Expresso algorithm infers the MSI status from RNA-seq FastQ files and will tag each set of FastQ file(s) as MSI-High (Microsatellite Unstable) or MSS (Microsatellite Stable).

Moreover, MSI Expresso can also provide an overview of MSI-related events such as exon skipping and unstable intronic and coding microsatellites represented as a CIRCOS plot and the associated raw count files allowing the identification of recurrent MSI-related events. As homopolymers are the most frequent microsatellites in the human genome (Subramanian et al., 2003) and are implicated in exon skipping, we chose to focus specifically on mono-nucleotide microsatellites of at least five nucleotides, referred as ‘microsatellite’ in the rest of the article.

#### Creation of a microsatellite database

We extracted a 60-bp sequence centered around every microsatellite located within a coding, UTR or intronic region of each transcript defined in NCBI RefSeq annotations (a given sequence can have multiple annotations due to the occurrence of multiple variants for a gene corresponding to the different protein isoforms). Moreover, in order to investigate potential relationships between flanking unstable intronic microsatellites and exon skipping events (skipping of one exon only), we extracted a 60-bp sequence at the junction of two consecutive exons, 30 bp at the 3′ end of the first exon and 30 bp at the 5′ start of the second exon, whenever a microsatellite was detected within 50 bp of the acceptor site of the first exon to identify reads that support the expected splicing of the transcript. Similarly, we also extracted 60 bp at the junction of the first exon and the exon following the second exon to identify reads supporting an exon skipping event.

Although we did not find any study mentioning a potential link between unstable microsatellites close to the donor site and exon skipping, we hypothesized that this could be the case as mutations in both acceptor and donor sites have been reported to cause exon skipping (Buhard et al., 2016; Jonchère et al., 2024; Lewandowska, 2013). Thus, we also extracted 30 bp at the 3′ end of the first exon and 30 bp at the 5′ start of the second exon whenever a microsatellite was found within 50 bp of a donor site.

MSI Expresso then performs an alignment of FASTQ files using BWA against the above custom made FASTA file and generates files of raw counts for each type of read: reads supporting exon skipping events vs. normal junctions, the microsatellite size distribution for each microsatellite located in a coding/UTR/intronic region.

#### MSI status inference

The MSI status estimation of a sample from MSI Expresso relies on the inferred MSI status of a custom panel of 192 UTR microsatellites (191 3′UTR and 1 5′UTR microsatellite). This panel of microsatellites has been established using RNA-seq data set of 101 normal/tumoral sample pairs of MSI-H CRC patients and 32 normal/tumoral sample pairs of MSS CRC patients, which were part of the MicroSplicother project (MICROSPLICOTHER - France Génomique) These microsatellites have first been chosen because they belong to house-keeping genes (Eisenberg and Levanon, 2013) and are thus expected to be expressed in most cells and tissues. Secondly, only microsatellites located in 3′UTR have been considered (except one located in 5′ UTR), as this approach should be compatible with every RNA-sequencing protocol, including those based on oligo (dT) priming-based RNA sequencing. At last, microsatellites with a length ranging from 15 to 25 bp were selected as they were supposed to be more unstable according to the polymerase slippage model (Daunay et al., 2019; Ellegren, 2004). Finally, they have been chosen for their quasi-exclusive instability in tumoral samples from the MicroSplicother project (“Microsatellite instability at U2AF-binding polypyrimidic tract sites perturbs alternative splicing during colorectal cancer initiation”, Jonchère et al., 2024).

The MSI status for one given microsatellite is given by the following formula:

$$S\left(m\right)=\log_{10}\left(\frac{\sum_{i\in M_{1}\left(m\right)}{\left(i\times r_{i}\right)}^{2}}{\sum_{i\in M_{0}\left(m\right)}r_{i}}\right)$$

Where m is a microsatellite belonging to the UTR panel, M_1 (m) = {r_i ∈R(m),i ≠0}with r_i,the number of reads covering m with a mapq ≥30, with a microsatellite size of i + L(m) where L(m) corresponds to the length of m on the reference sequence and r_i/N ≥ 10%, N being the total number of reads covering m and R(m) = {r_i, ∀i∈ℤ} and M0(m) = M_1 (m)∪{r0}.

If S(m) ≥ 0.75, the microsatellite status is MSI-H, for 0.75 > S(m) ≥ 0.2, the status is MSS otherwise. The MSI status M for one given sample is calculated only if the overall percentage of expressed microsatellites within the panel is over 30% and is determined by the percentage, pH, of the expressed microsatellites (coverage ≥15 and at least five reads carrying a mutated microsatellite) belonging to the UTR panel predicted to be MSI-H as follows: M = MSI-H if pH > 35%, M = MSS otherwise. These different cutoff values, customizable by the user, have been set empirically using the MICROSPLICOTHER data set and have been shown to work well for all the tested datasets. The individual instability status of each microsatellite (coding, UTR or intronic) found in RNA-Seq sequences is determined by the above calculation score. For the skipping events, a reduced version of this score is computed with i having a fixed value of five considering a possible degradation of the mRNA presenting the skipping event by the nonsense mediated decay system. By doing this, we can keep the same score cutoffs for unstable microsatellites and skipping events.

### Overview of MSI-related events

On top of the MSI status inference that could be useful in a clinical setting, MSI Expresso also offers a visualization across a group of samples for three types of events: exon skipping events for which a microsatellite is present within 50 bp of the donor or acceptor site of the first exon involved in the skipping, unstable coding and intronic microsatellites. This type of representation along with their equivalent tabular files could be particularly helpful for researchers in order to identify potential recurrent mechanisms among a cohort of patients.

For exon-exon junctions, spanning reads to be considered in the counting must overlap totally one side of the junction sequence (30 bp) extracted in the “Creation of a database of microsatellites” section and a minimum of eight bp on the other side. An exon skipping event is considered interesting if a microsatellite is present within 50 bp of the donor or acceptor site of the first exon involved in the skipping and Sk(m) ≥ 0.75 with rs the number of exon skipping supporting reads and Sk(m) defined as:

$$S_{k}\left(m\right)=\log_{10}\left(\frac{{\left(5\times r_{s}\right)}^{2}}{\sum_{i\in M_{0}\left(m\right)}r_{i}}\right)$$

Similarly, MSI Expresso considers unstable coding and intronic microsatellites (coverage ≥15 and at least five reads carrying a mutated version of the associated microsatellite). Reads carrying a microsatellite are only retained if they contain the entire sequence of the microsatellite. Using this selection of events, MSI Expresso displays the associated exon skipping events and unstable coding and intronic microsatellites in a Circos plot facilitating the identification of potential recurrent events for the samples of interest.

All these parameters can be changed by the user in the configuration file and the MSI status of each microsatellite and sample can then be re-evaluated without having to repeat the entire analysis. The analysis script only requires a configuration file as an argument. Configuration parameters optionally allow the output of a detailed file with the name and the type of each matching read and a bam alignment file containing each read carrying a microsatellite or presenting an MSI-related event, dramatically reducing the disk space required.

We provided the script MSIspecific.pl for selecting MSI specific recurrent events (MSI coding, intronic and UTR microsatellites and skipping events) across a set of samples whose status (MSI or MSS) is determined by MSI Expresso. We also provide the MSI Expresso2circos.pl perl script to easily generate a CIRCOS image representing the status of a set of microsatellites/skipping events (typically: recurrent events found with MSIspecific.pl script) for a set of samples as shown in Figure 2.

### MMRd status estimation

As we know the MMRd status (mismatch repair deficiency) of some tested public samples -out of the MICROSPLICOTHER project-we have developed a very simple tool to estimate the loss of expression of the three major genes involved in the mismatch repair system for small indels: *MLH1*, *MSH2* and *MSH6*. The most frequent event in MMRd cancers is the *MLH1* promoter hypermethylation (MLH1ph) (Pannafino and Alani, 2021). We did not include the *PMS2* gene in this test, since we always found very high expression levels for this gene - even in MSI-H samples. Despite being part of the MMR system for short indels, *PMS2* dysregulation is a very rare event in colorectal cancer (Moreno et al., 2020). Even if *MSH6* loss of expression is not frequent (Moreno et al., 2020), we used it in our test as we find one MSI sample with low expression, and *MSH6* expression level in normal samples - slightly lower than the two other tested genes-make it a reliable expression reference as well as *MLH1* and *MSH2*.

**FIGURE 3:**
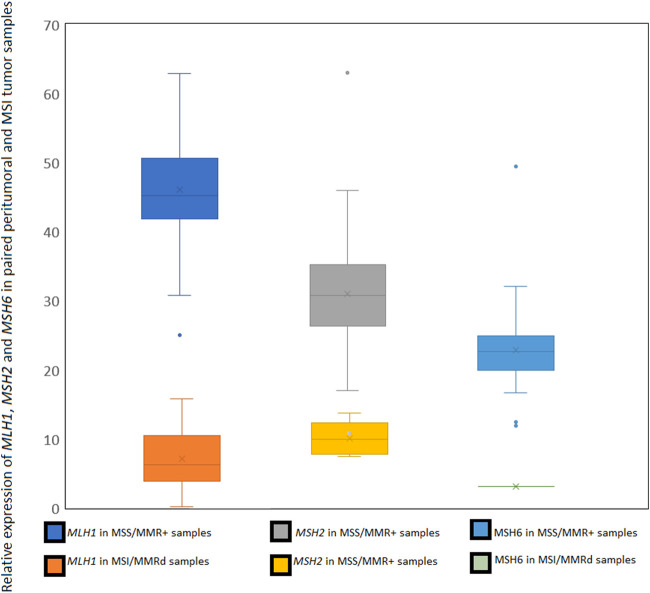
Specificity of the selected microsatellites panel for MSI tumoral samples in MICROSPLICOTHER dataset.

By comparing the relative expression of each one of these genes, we were able to confirm the known MMRd status caused by the knock-out of one of the two main genes: *MLH1* or *MSH2*, in accordance with the MSI-H status. A simple normalization is applied to read counts, to take in account the length of each CDS. In normal cells, we observe a relative expression of each of these three genes is close to 35% ( ± 10%), in MMRd samples this percentage drops below 15% for *MLH1* or *MSH2* genes and below 10% for *MSH6* (Figure 3). We also provide a variant call file (vcf) for MMR genes *MLH1*, *MSH2*, *MSH6* and *PMS2* for potential functional mutations and a bam alignment file for visualization of read mapping. The user can add genes to the functional variant calling by adding the CDS sequence to the reference file (see MSIExpresso documentation for details).

## Results

### The MICROSPLICOTHER dataset

**FIGURE 4:**
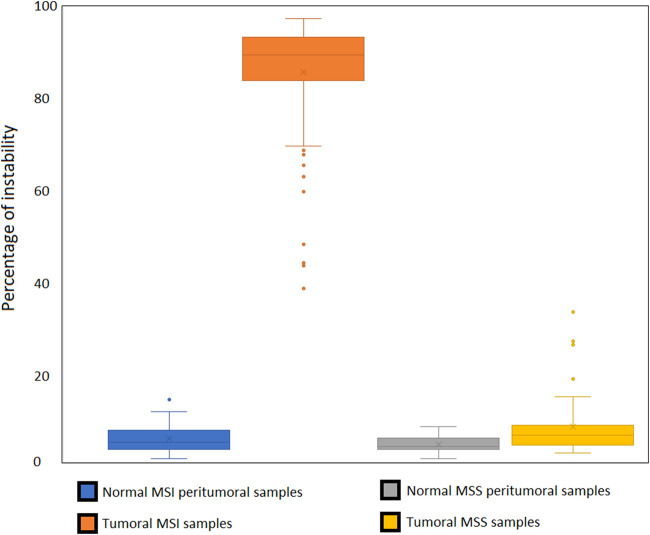
Relative expression of *MLH1, MSH2* and *MSH6* in MSS/MMR + normal samples compared to MMRd/MSI samples.

This dataset was used to develop MSI Expresso software, determining MSI specific events by comparing instability in MSS and MSI tumoral pairs and cell lines (Figure 4). All pairs of peritumoral and MSS tumor samples were correctly identified as MSS. Among the 75 pairs of peritumoral and MSI tumor samples, seven tumors were identified as MSS and three normal peritumoral samples as MSI. Among them, three pairs of peritumoral and tumor samples were swapped in their MSS/MSI status, suggesting an inversion between tumor and normal sample. MSI samples identified as MSS could also be due to an incorrect initial assessment. Considering only one normal sample with an MSI status, the false positive rate for this study is approximatively 1%. Except for one sample, the *MLH1*, *MSH2* and *MSH6* loss of mRNA expression is always in concordance with the sample MSI status. The full status for the 287 tested samples is in Supplementary Table S1.

### Microsatellite panel relevance

**FIGURE 5:**
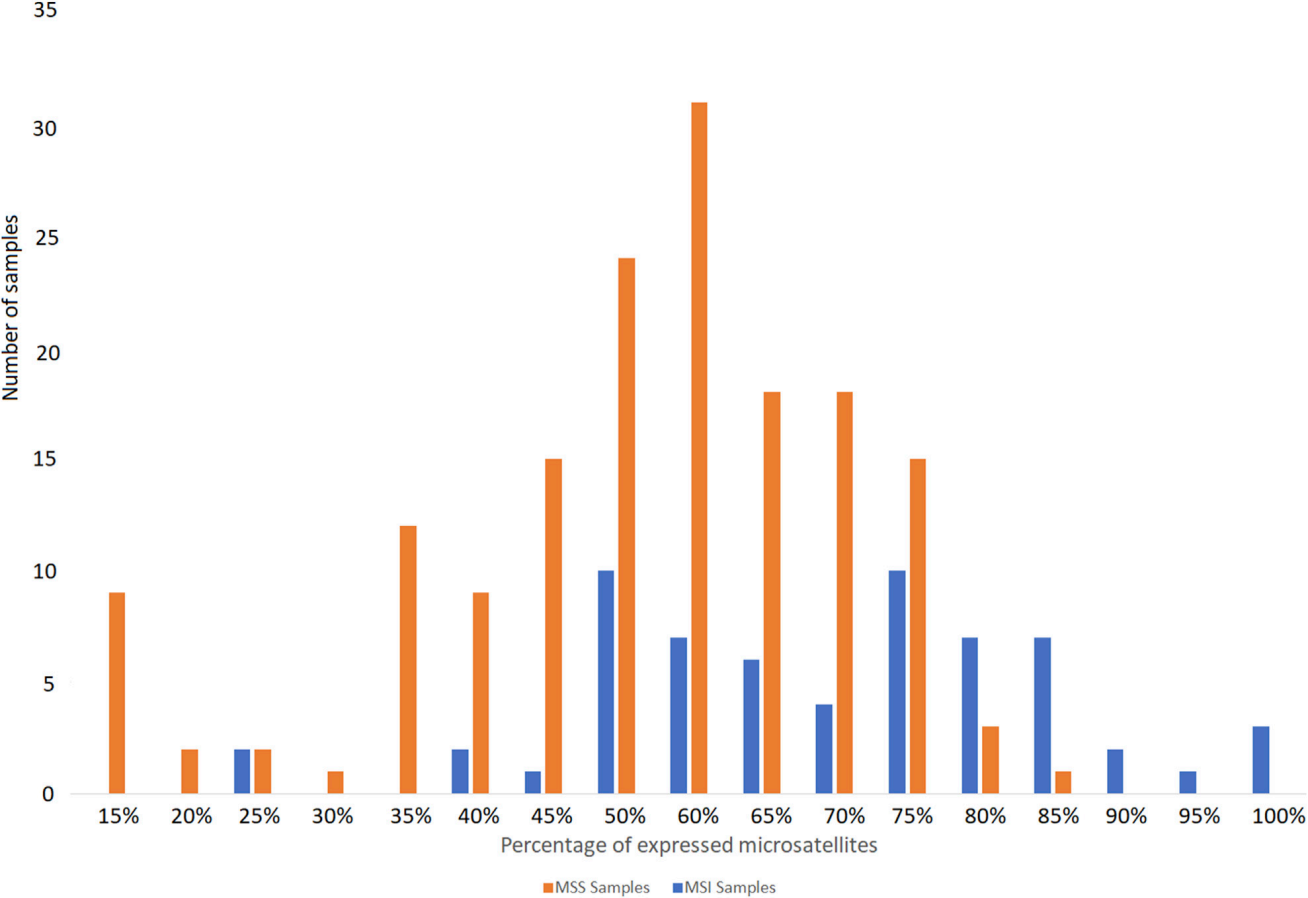
Percentage of microsatellites of the panel found expressed in the publicly available RNA-seq data tested from MSS and MSI cancer samples.

We designed our reference panel to be applicable to various tissue types, ensuring a sufficient number of expressed microsatellites to accurately estimate MSI status. Therefore, most of the tested samples expressed more than 60% of the 192 microsatellites included in the panel (Figure 5).

**FIGURE 6:**
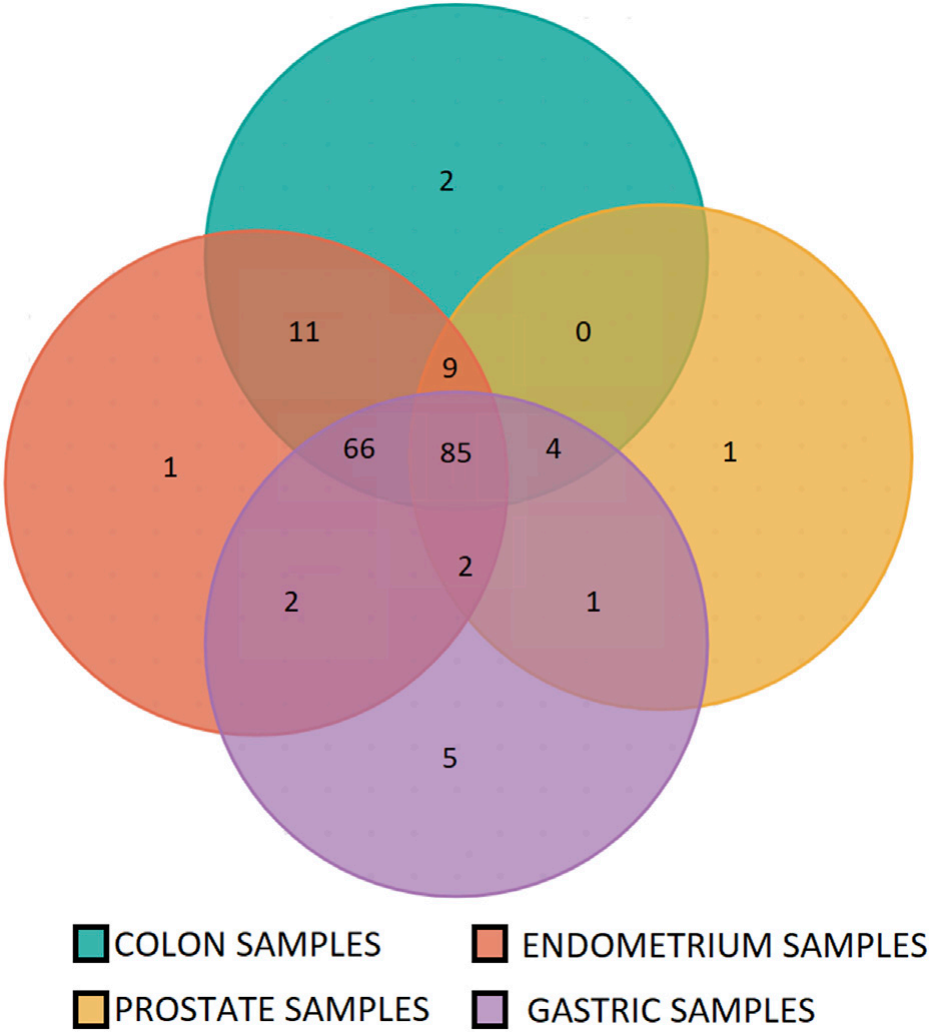
Specificity of the microsatellites panel for colon, stomach, endometrium and prostate tumor samples. 86% of the panel microsatellites are found instable in at least three of four tested tissues (166 out of 192 panel microsatellites).

The microsatellite panel was designed using MSI colorectal cancer samples. To ensure that this panel was not tissue-specific, we tested it on endometrium, gastric and prostate cells lines, peritumoral and cancer samples with known MSI status. Only a few of the most frequently expressed microsatellites were specific to the colon (Figure 6). A detailed list is given in Supplementary Table S2.

### Assessment of MSI status on freely available NGS RNAseq cancer data

We analyzed RNASeq FASTQ files containing reads from 50 bp to 150 bp in length from gastrtric cancers (Min et al., 2016; Yoon et al., 2013), prostate (Ju et al., 2018; Sandoval et al., 2018; Thomas-Jardin et al., 2018), 18 colorectal cancer (Kaviyarasan et al., 2024) and endometrial cancer (Ghandi et al., 2019; Tsukamoto et al., 2014) samples and cell lines publicly available from NCBI SRA and EBI ENA using MSI Expresso.

We confirmed the published MSI status of the 109 samples, whose MSI status was assessed in their original studies using DNA (Supplementary Table S3).

**FIGURE 7:**
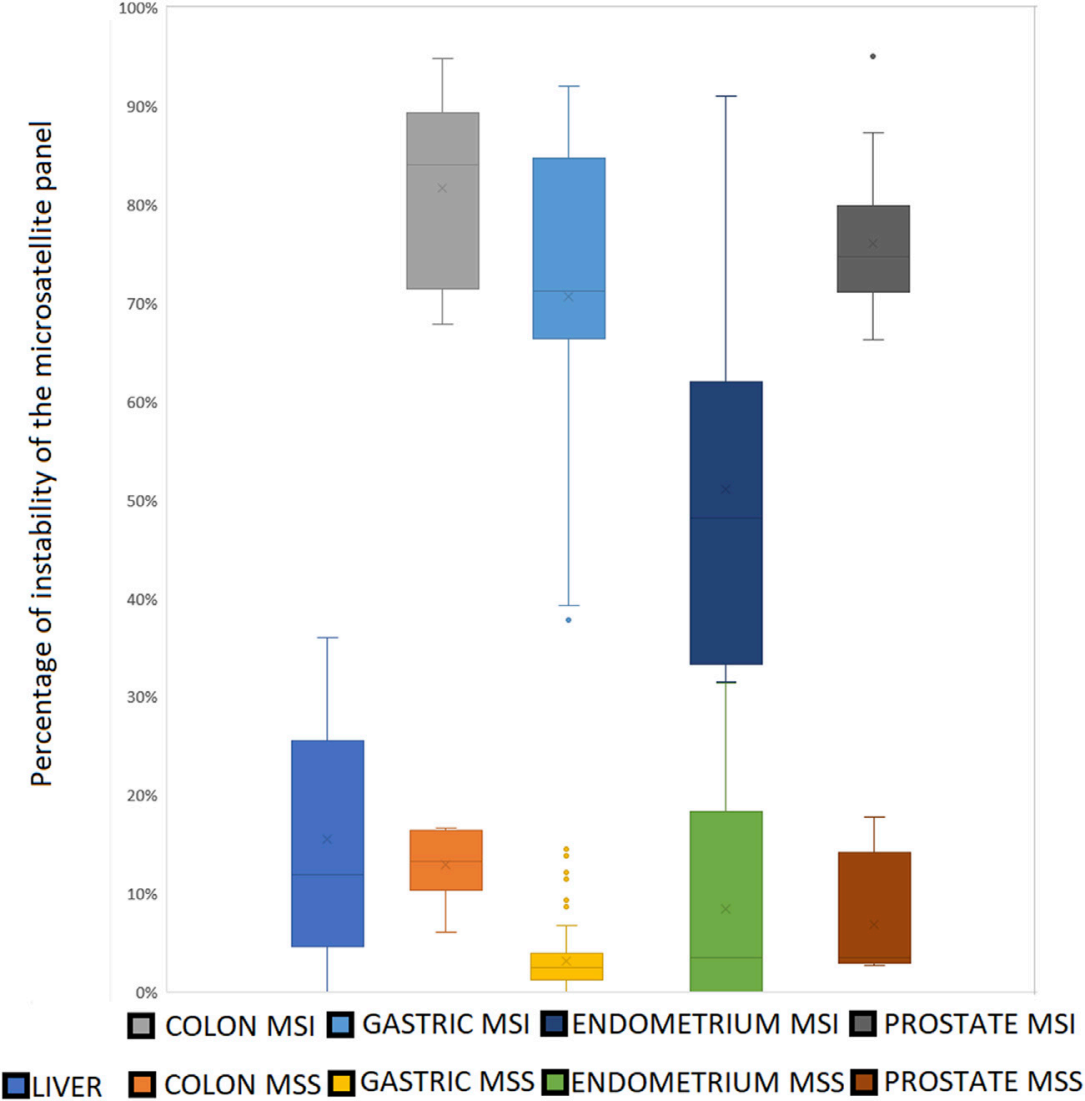
The clear cut between MSS and MSI status using our microsatellite panel. Very few MSI samples show an instability ratio under 50%. All the MSS samples present an instability ratio below 15% in mean coverage datasets. Even if mean percentages vary between tissues, there is still a gap between MSS and MSI samples for a given cell type.

**FIGURE 8:**
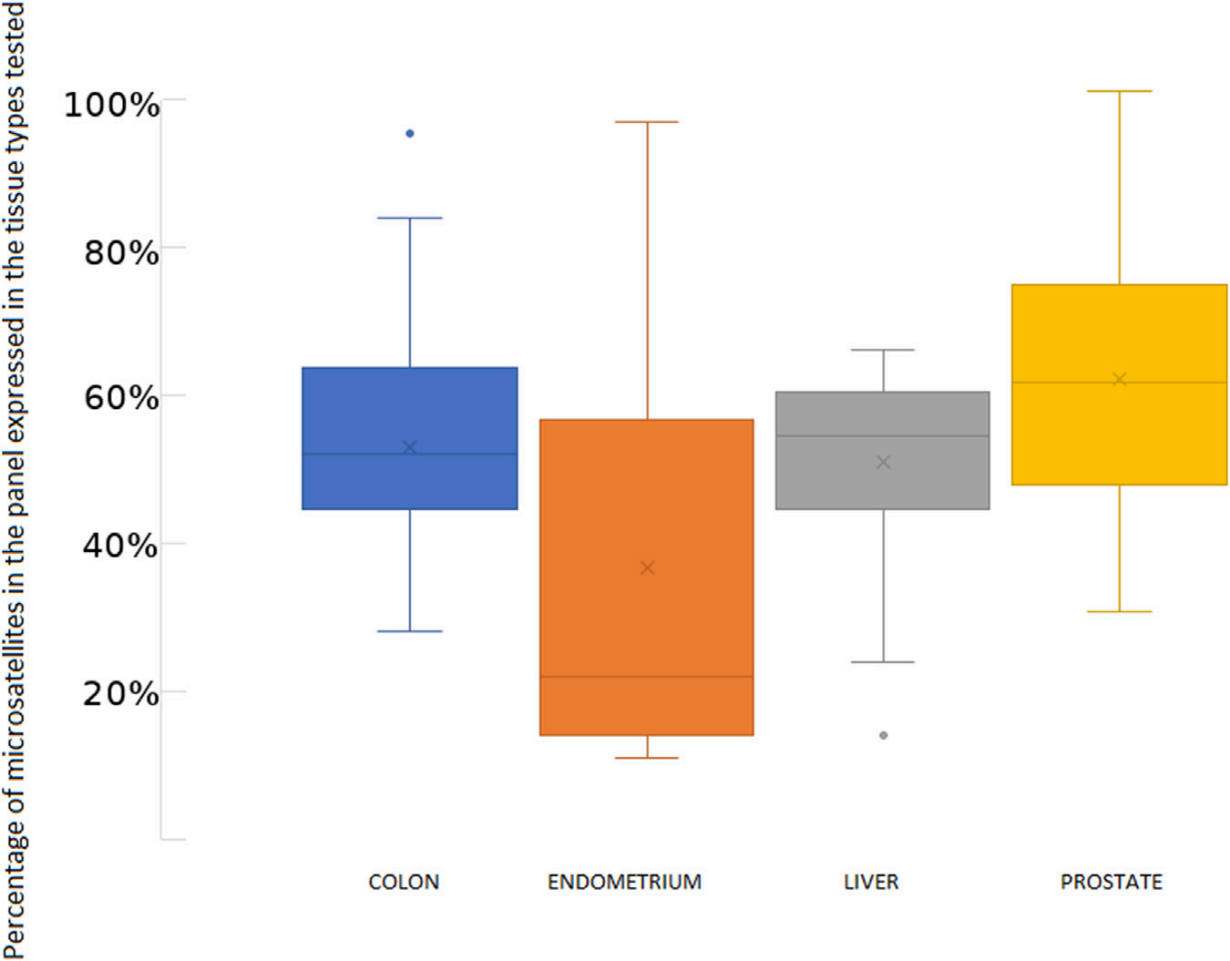
Panel microsatellites expression across tested samples. Note that endometrium samples with less than 40% of expressed microsatellites present a low coverage (<25M of reads per sample).

− 21 MSI-H samples (15 gastric samples from 2 Korean studies, 13 prostate cell lines LNCaP replicates, MDAPCA2B, 13 22Rv1 replicates and DU145, endometrial cell lines HEC1B and AN3CA, large intestine cell lines SW48 and LS180) with 38%–95% of instability,
− 87 samples (75 gastric samples from 2 Korean studies, 6 VCaP prostate cell line replicates, breast cancer cell line MCF-7, three pairs of normal and tumoral endometrial samples) with 0%–17% of instability in the MSS samples (Figures 7, 8)

We noted a potential inversion between normal and tumoral samples r18-917N and r18-917T (NCBI SRA runs SRR801432 and SRR801433) also suggested by the MMRd status of both samples. In MSI-H samples, we also confirmed the presence of frameshifts in the coding sequence of the *ACVR2A*, *TGFBR2* and *BAX* genes due to a change in the size of a coding repeat, and the skipping of exon nine of *HSP110* in association with the observed shortening of a T17 microsatellite at two nucleotides from the acceptor site. Other skipping and coding events were specifically detected in several MSI samples at various frequencies (Supplementary Table S4). We were able to analyze a great number of intronic microsatellite due to the presence of reads from unspliced RNAs carrying polyA intronic microsatellites retained by the polyA + selection when this method was used as RNA capture (Svoboda et al., 2022).

In order to estimate the false positive rate of our algorithm, we analyzed a dataset of 34 paired hepatocellular carcinoma samples (SRP338575 study), this type of cancer having very low frequency of MSI (Mukai et al., 2021). One tumoral sample showed an MSI status just above the 35% cutoff of instability, and none of the normal samples were found MSI-H, we estimate that without any change to the default MSIExpresso parameters, the false positive rate (FPR) is about 3%, by refining the microsatellite panel for a particular analysis or changing the MSS/MSI cutoff, the FPR could be lowered.

### MMRd status estimation

Most of the MSS samples tested have a ubiquitous expression of the three main MMR genes known to be (epi)mutated in MMRd cancers. 3 MSS colon samples presented a low relative expression of *MLH1* or *MSH6* (close to the 15% cutoff), whereas MSI samples show very frequent under-expression of *MLH1*. In the case of the cell line LNCaP we confirm the loss of expression of *MSH2* in every tested sample. We also found a loss of expression of *MSH6* in one MSI endometrial cancer sample. The 22rv1 cell line showed no loss of expression in tested samples from the same study, whereas this cell line is known for its MLH1ph phenotype, no structural variant was found in all the MMR tested genes. None of the tested genes show any expression dysregulation.

## Discussion

Being able to determine the MSI status of a tumor sample is crucial in oncology, especially for solid cancer patient management due to the significant amount of prognostic and predictive information carried by this alteration. Thus, the MSI status is particularly important for the administration of immune checkpoint inhibitors to cancer patients bearing this genetic alteration in their tumor (Marcus et al., 2019), and is therefore crucial for therapeutic decision-making. In our study, we developed a bioinformatic tool, MSI Expresso, which gives a general overview of MSI in RNASeq data with an accurate estimation of the MSI status using a panel of 192 3′UTR microsatellites expressed in housekeeping genes and provides a refined snapshot o. f the subsequent MSI-related events. Although other algorithms for MSI detection based on transcriptomic data have been described (Danaher et al., 2019; Lu et al., 2013; Pačínková and Popovici, 2019), MSI Expresso is, to our knowledge, the first publicly available ready-to-use software which can infer the MSI status from RNA-seq data.

Furthermore, the output files of MSI Expresso (raw event counts) may be used in new bioinformatic approaches such as machine learning and deep learning to characterize MSI related pathologies beyond CRC.

Our approach is based on a panel of 3′UTR microsatellites and presents the following advantages:

These non-coding microsatellites are not a nonsense-mediated mRNA decay target despite their instability and they should thereby be abundant in MSI samples as well as in MSS samples. These microsatellites are significantly longer than the coding microsatellites and are therefore more prone to instability. They are abundant among housekeeping genes and provide a large panel for testing instability across tissues.

MSI Expresso does not require paired normal/tumoral data as naturally unstable microsatellites can be filtered out using any public dataset of RNASeq experiments of the same tissue with equivalent coverage. Our approach has similarities with the one developed by (Lu et al., 2013), as it also considers microsatellites insertions and deletions. However, we used a more targeted approach as we analyzed a small panel of 140 microsatellites compared to all microsatellites in the former study (Lu et al., 2013). The machine learning approaches proposed by (Danaher et al., 2019; Pačínková and Popovici, 2019), are based on the expression of a panel of selected genes. In (Danaher et al., 2019) study, the selected genes are assumed to be expressed in pancancer but they presented a quite high false-positive rate (≥20% on the COAD, STAD and UCEC cohorts). In (Pačínková and Popovici, 2019) study, the gene list was built for colon cancer and thus showed better results with this cancer type than with others (AUC = 0.94, 95% CI = (0.90–0.97) with colon cancer samples, AUC = 0.90, 95% CI = (0.85–0.94) with gastric cancer samples and AUC = 0.71, 95% CI = (0.62–0.81) with endometrial cancer samples).

MSI Expresso was further validated in four cancer types known to present MSI phenotype with variable frequencies: gastric (≈40%), colon (≈20%), prostate (<5%) and endometrium (≈60%) (Hause et al., 2016) confirming the reported MSI status in the associated public datasets. Our results showed 99% of concordance for the MSI status in CRC, endometrial and prostate samples. The only discordant status was observed in one Chinese CRC MSI sample (2 replicates) assessed as MSS, falling slightly below the 33% MSI/MSS threshold (32.4% of instability for this sample).

MSI Expresso also includes the identification of exon skipping events caused by microsatellite instability. This analysis may allow the discovery of specific alternative transcripts expressed in various cancers beyond the assessment of MSI status. The identification of such events could be crucial for both basic research and clinical oncology, as exemplified by the well-known mutant HSP110 protein in MSI colorectal cancer, which arises from exon skipping cause by a deletion in a T17 microsatellite (Dorard et al., 2011).

The following are some limitations encountered with MSI Expresso. RNA sequencing data are generally less frequently available for tumor sample and the MSI status is not always known for the freely available RNASeq experiments (NCBI SRA, EBI ENA), limiting drastically the ability to extensively test MSI Expresso on a broad set of samples in various normal and tumoral tissues. Although we used widely expressed genes in our panel, MSI status may be difficult to assess for samples with low coverage (less than 25 M reads) as a fewer number of microsatellite sequences would be available. See Figure 4 where endometrium samples with 23 M reads express less than 30% of the microsatellite panel.

We provide a large panel of microsatellites to assess the MSI status, depending on the RNA sequencing depth, some of these microsatellites may appears unstable across MSS and MSI samples. MSI Expresso was initially developed and tested on Illumina HiSeq 2000 101-bp paired-end RNASeq assays. With the evolution of NGS sequencing platforms over the years and the increase of publicly available RNASeq data for both MSS and MSI samples, we have recently had the opportunity to assess our software using different technologies and coverage depths. On the latest platforms with higher coverage, we noticed an increased level of instability in the MSS samples, while this level remained unchanged in the MSI samples, thereby narrowing the distinction between MSI and MSS status.

Our method for estimating MMR status is designed as a simple check for the major known MMRd events such as MLH1 and MSH2 loss of expression. This method allows us to correlate 60% of MSI-H status with MMRd status and over 99% of MSS status with MMR + status. Samples with a known MLH1ph status are always consistent with our analysis. An exhaustive search for events responsible for MMRd status, such as full RNA-Seq variant calling and global differential expression analysis are beyond the scope of our software. Pipelines for these analyses are already available, but would require paired normal/tumoral samples.

## Conclusion

In conclusion, MSI Expresso is an easy-to-use and accurate tool for estimating the MSI status and related MMR status from RNAseq data of tumor samples. It also allows the identification of several MSI-related transcription events from RNA-seq data from tumor samples. It could thus be useful both in a clinical setting for precision oncology by helping tailoring cancer treatment for solid cancers and for basic research for the understanding of cancer biology and pathogenesis.

## Data Availability

The original contributions presented in the study are included in the article/Supplementary Material, further inquiries can be directed to the corresponding author.
